# Effects of testosterone on gene expression in males and females across 40 human tissues

**DOI:** 10.1038/s41598-026-40863-2

**Published:** 2026-02-23

**Authors:** Evans Kiptoo Cheruiyot, Zhu Zhihong, Allan F. McRae

**Affiliations:** 1 Institute of Molecular Bioscience, The University of Queensland, Brisbane, QLD Australia; 2 National Centre for Register-Based Research, Aarhus University, Aarhus V, 8210 Denmark

**Keywords:** Sex difference, Gene expression, Testosterone, GTEx, UK Biobank, GWAS, Gene expression, Gene regulation

## Abstract

Variation in testosterone levels is associated with pronounced health risks, often in a discordant manner between males and females. While studies have demonstrated a sex-specific genetic architecture for testosterone, the biological basis for the differential impact on diseases between the sexes is largely unknown. In this study, we correlated predicted testosterone and within-sex gene expression measures across 40 human tissues to identify genes that show sex-differential control of gene expression and examine how this varies across tissues. Gene expression measures were obtained from the Genotype-Tissue Expression project (v8 GTEx release), with sex-specific genome-wide summary statistics from the UK Biobank used to construct polygenic scores as proxies for total testosterone and bioavailable testosterone. We quantified the proportion of variance in the genomically predicted testosterone levels that was captured by gene expression measures within each sex/tissue pair. The association between predicted testosterone measures was tested within each sex/tissue pair for over 20,000 gene transcripts. Gene expression levels in the mammary breast (R^2^ = 0.54 ± 0.20), adipose (visceral omentum; 0.56 ± 0.21), esophagus (muscularis; 0.42 ± 19) and skin (sun exposed lower leg; 0.34 ± 0.16) tissue explained the largest proportion of total testosterone variance in females, although these estimates were not significant after correction for multiple testing across tissues. Association analyses identified significant associations between total testosterone and gene expression across four transcripts (*NUPR1L*, *PTPRD*, *PSPHP1*, and *RP11-208G20.3*) in the skeletal muscle, tibial artery, and pancreas tissues for females p < $\:2.37\times\:{10}^{-6}$, whereas no significant associations were observed in males. No transcript or gene showed a significant association with predicted bioavailable testosterone across all study tissues in both males and females. The relationship between testosterone and gene expression levels is complex, showing variation across tissues and between the sexes. While we found limited numbers of associations between individual gene expression levels and predicted testosterone levels, our whole transcriptome approach found that a substantial proportion of testosterone levels could be captured by gene expression levels, indicating that more associations could be identified with larger sample sizes and directly measured hormone levels.

**Supplementary Information:**

The online version contains supplementary material available at 10.1038/s41598-026-40863-2.

## Introduction

Sex differences in complex traits and diseases are widespread in humans. The majority of the genetic control of human complex traits is found on the autosomal chromosomes^1^; however, autosomal genome profiles (i.e., DNA sequence, gene structure, allele frequencies) do not differ between males and females^2^. Indeed, autosomal genetic correlations between males and females are close to one across human quantitative traits and disorders^3^. However, the autosomal gene regulation functions in a sex-specific manner to cause phenotypic differences between the sexes^2^. Gene regulation mediated by sex hormones is a major driver of these sex differences by directly influencing the traits^2,4^ or acting as an amplifier in a sex-biased manner^5^.

Testosterone (TT), one of the essential sex hormones, is primarily produced by the testes in men and the ovaries and adrenal glands in women. Men have a higher level of circulating testosterone than women^6^. About half (~ 40% in males, ~ 60% in females) of the circulating testosterone is tightly bound to sex hormone-binding globulin (SHBG), while a smaller fraction (~ 40–50%) is loosely bound to serum albumin, and together with the free or unbound testosterone (~ 1– 4%), constitute bioavailable (BioT) testosterone^7–9^.

Although still debated, BioT is often considered the biologically active component of circulating testosterone and could be more directly linked to transcriptional regulation^9–11^. Nonetheless, TT has been shown to be a stronger predictor of morbidity and mortality in men than free testosterone in some studies^12,13^, suggesting that total testosterone may also capture biologically meaningful trait variation. Notably, there is still a lack of consensus on the clinical utility of TT versus BioT. Some organizations, such as the Australian Endocrine Society^14^, recommend TT for clinical decision-making, whereas recent studies^15^ suggest that BioT measurements provide additional clinical advantages over TT alone. Overall, these reports suggest that TT and BioT may capture distinct but complementary aspects of testosterone biology.

Serum testosterone is a highly heritable phenotype, with a heritability estimates of around 60% in men and 40% in women from pedigree studies^16,17^. However, the genetic correlation for testosterone levels between males and females is close to zero, suggesting distinct biology of testosterone between the two sexes^3,18^. This pattern has been linked to differences in testosterone regulation, with a greater role of the hypothalamic-pituitary-gonadal (HPG) axis in males and stronger SHBG involvement in negative feedback regulation compared to females^6^.

Testosterone is associated with various human traits and disorders^3,8^, although the relationship can differ in males and females. For example, higher testosterone levels are associated with lower risks of type 2 diabetes and stroke in men, but higher risks in women^3,8^, although evidence for causality remains inconsistent across these studies. The genomic mechanisms of the sex differences in the relationship between disease and testosterone remain largely unknown. The relationship between testosterone and disease becomes more complex as testosterone can affect gene expression in a tissue-specific manner. For example, studies on animal models suggest that testosterone acts primarily on the liver transcriptome to cause metabolic-related disorders, including type 2 diabetes^4^. Understanding the associations between testosterone and gene expression across tissues could provide insight into testosterone role in sex specific traits or disease risk.

In this study, we utilized publicly available sex-stratified GWAS summary statistics for total^6^ and bioavailable testosterone levels^3^ to construct sex-specific polygenic scores (PGS) for these traits in the Genotype-Tissue Expression (GTEx) samples^19^. We then correlated the predicted PGS with gene expression measures across 40 human tissues to investigate whether the patterns of association differ between men and women. Additionally, we performed transcriptome-wide association studies (TWAS) to identify testosterone-responsive genes within each sex–tissue pair. Together, these findings provide insights that may guide molecular experiments and contribute to understanding the biological basis of sex-specific testosterone effects.

## Materials and methods

### GWAS summary data

We downloaded sex-specific genome-wide association summary statistics for the total testosterone (TT)^6^ and bioavailable testosterone (BioT)^3^. Both studies employed similar methodologies for conducting the GWAS.

Briefly summarising the methodology in these studies, the sex-stratified GWAS summary data for total testosterone^6^ was derived from a subset of the ‘White British’ cohort from UK Biobank^20^. After quality control and pre-correction, the analysis included 146,339 males and 142,778 females, with 15,221,426 SNPs in males and 15,359,595 SNPs in females. Similarly, the sex-specific GWAS for BioT^3^ was performed using a subset of the ‘White European’ cohort from the UK Biobank. After quality control, the analysis included 178,782 males and 188,507 females, with 16,577,424 SNPs in males and 16,585,745 SNPs in females.

Before running the GWAS, the raw TT measurements were log-transformed and pre-corrected as detailed in^6^. The covariates included 40 principal components (PCs), age, sex, 5-year age ⋅ sex, self-identified ethnicity, self-identified ethnicity ⋅ sex, fasting time, assessment centre, genotyping batch, estimated dilution factor, time of sampling, and day and month of assessment. The GWAS for adjusted TT was performed using PLINK v2.0^21^ for males and females separately using the following command: [plink2 –bfile file –glm –geno 0.2 –hwe 1e-50 midp –out file].

BioT was calculated from TT using the Vermeulen equation, accounting for measured SHBG and albumin concentrations, as described in^3^. BioT values were then adjusted separately for males and females. For males, BioT was inverse normal transformed and adjusted for fasting time, age at baseline, assessment centre, genotyping chip, genetic data release, and 10 PCs. In females, BioT was log-transformed and adjusted for age at baseline, dilution, batch, time since blood draw, blood draw time, menopause status, operation status, and 10 PCs. The GWAS of adjusted traits was conducted for autosomal SNPs (MAF > 1%) using BOLT-LMM in males and females, separately.

### GTEx data

We used GTEx release version 8 RNA-seq dataset (dbGaP Accession phs000424.v8.p2) in analyses. This dataset included preprocessed, normalised, and filtered gene expression values across 54 tissues for 838 individuals and genotype data. The majority of donors were of European American ancestry (*N* = 715; 85.3%), followed by African American (*N* = 103; 12.3%), with Asian American, Hispanic, and Latino individuals comprising approximately 1–2% of the cohort^19^. A total of 557 donors were male (mean age = 53.5 years; IQR = 47–62; range = 20–70) and 281 were female (mean age = 51.9 years; IQR = 44–63; range = 21–70). The quality control (QC) procedures are documented in the GTEx portal (URLs) and in^19^. Briefly, for each tissue, gene expression was quantified based on the GENECODE 26 annotation (URLs). TMM (Trimmed Mean of M-values) method in edgeR^22^ was used to normalise raw gene counts. Genes were restricted to those with ≥ 6 reads and TPM (transcript per million) > 0.1 in ≥ 20% of the samples. The expression values for each gene were then normalised using a normal inverse transform across samples. The expression values were adjusted for possible covariates, including PEER [probabilistic estimation of expression residuals] factors to account for hidden batch effects, the top 5 genetic PCs, the genotyping platform and protocol, and sex as described in^19^. The top 5 PCs was found to significantly correlate with population substructures in the GTEx data^19^.

We restricted our association analyses to tissues with at least 30 samples in each sex, resulting in 40 tissues found in both sexes and five sex-specific tissues (testes, ovary, prostrate, uterus and vagina) remained for analyses. The number of samples per tissue in the final gene expression files ranged from 78 (brain spinal cord cervical c-1) to 469 (muscle skeletal) in males and 33 (brain substantia nigra) to 237 (skeletal muscle) in females. Each tissue contained expression information for over 20,000 transcripts (see Additional file 1: Table S1).

### Polygenic prediction of testosterone (PGS_T_)

The GTEx dataset does not include direct measurements of testosterone for the samples. Therefore, to investigate the association between testosterone and gene expression using GTEx data, we calculated polygenic scores (PGS) for each sample using the SNP weights from the above-described UK Biobank summary statistics for testosterone traits. We estimated SNP weights using the SBayesR software^23^, assuming initial mixture probabilities (π = 0.95,0.02,0.02,0.01) and 10,000 iterations, of which 2000 were discarded as burn-in in all analyses. We used a banded reference LD matrix constructed from a random set of 10,000 unrelated UK Biobank individuals of European ancestry^23^. We generated PGS for each individual in the GTEx dataset using PLINK v1.9^21^ for males and females separately:

1 $$\:PGS=\frac{{\sum\:}_{i}^{N}({S}_{i}\times\:{G}_{ij})}{2{M}_{j}}\:$$

where $\:{S}_{i}$ = the weight for SNP $\:i$; $\:{G}_{ij}$ = coded genotype of SNP $\:i$ in individual $\:j$; $\:{M}_{j}$ = number of non-missing SNPs in sample $\:j$. Hereafter, we will refer to genetically predicted testosterone and BioT as PGS_T_ and PGS_BioT_, respectively.

### Omics restricted maximum likelihood analyses (OREML)

The proportion of variance ($\:{R}^{2}$) for PGS_T_ or PGS_BioT_ captured by the genome-wide gene expression transcripts was estimated using restricted maximum likelihood (OREML) based on OSCA (OmicS-data-based Complex trait Analysis) software^24^ for each sex and tissue, separately. We included age and body mass index (BMI) to account for potential confounding, as both age and BMI have known associations with testosterone levels^18,25,26^:

2 $$\:\mathbf{y}=\mathbf{C}+\mathbf{W}\mathbf{u}+\mathbf{e}$$

where $\:\mathbf{y}$ is an $\:n\times\:1$ vector of PGS_T_ or PGS_BioT_; $\:n$ = number of samples in each tissue (see Additional file 1: Table S1); **C** = quantitative covariates; **W** = $\:n\times\:m\:$matrix of $\:m$ gene expression transcripts (see Additional file 1: Table S1); is a $\:2\times\:1\:$vector of fixed effects; $\:\mathbf{u}$ is an $\:m\times\:1\:$vector of joint effects of all transcripts on predicted testosterone [$\:\boldsymbol{u}$ ~$\:\:N(0,\:{\mathbf{I}\sigma\:}_{u}^{2})$]; $\:\boldsymbol{e}$ = vector of random residuals [$\:\boldsymbol{e}$ ~$\:\:N(0,\:{\mathbf{I}\sigma\:}_{e}^{2})$]. The variance (***y***) = **V** = $\:W{W}^{{\prime\:}}{\sigma\:}_{u}^{2}+\:{\mathbf{I}\sigma\:}_{e}^{2}$ = $\:\mathbf{A}{\sigma\:}_{o}^{2}+\:{\mathbf{I}\sigma\:}_{e}^{2}$ with **A** = $\:W{W}^{{\prime\:}}/m$ and $\:{\sigma\:}_{o}^{2}$ = $\:{m\sigma\:}_{u}^{2}$. **A** is the omics-data relationship matrix (ORM) computed as:

3 $$\:{A}_{jk}=\:\frac{1}{m}\sum\limits_{i}\frac{\:({x}_{ij}-{u}_{i})\left({x}_{ik}-{u}_{i}\right)}{{\sigma\:}_{i}^{2}}$$

where $\:{A}_{jk}$is the relationship between individual *j* and *k*; $\:{x}_{ij}$is the unstandardized gene expression level of transcript *i* in individual *j*; $\:{u}_{i}$ and $\:{\sigma\:}_{i}^{2}$ are the mean and variance of transcript *i*, respectively; and $\:m$ is the number of transcripts per tissue (see Additional file 1: Table S1).

The $\:{R}^{2}$ for PGS_T_ or PGS_BioT_ was calculated as: $\:{R}^{2}=\:{\sigma\:}_{o}^{2}/({\sigma\:}_{o}^{2}+{\sigma\:}_{e}^{2}$). Variance-component p-values were adjusted for multiple testing across tissues using the Benjamini–Hochberg false discovery rate (FDR) procedure.

### Mixed-linear-model omic association (MOA)

We used the MOA approach implemented in OSCA^24^ to test the association between each gene expression transcript and the predicted sex hormone levels across all the study tissues. We included age and BMI as fixed covariates in the statistical model, as described earlier:

4 $$\:\mathbf{y}={\mathbf{w}}_{i}{b}_{i}+\:\mathbf{C}+\mathbf{W}\mathbf{u}+\mathbf{e}$$

which is and extension of model (2) above with an addition of new terms: $\:{\mathrm{w}}_{i}$ is an $\:n\times\:1$ vector of the *i*-th gene expression; and $\:b$ is the effect of gene transcript *i*. Definitions of the remaining parameters are provided above. We used Bonferroni correction (0.05/$\:m$; where $\:m$ is the number of transcripts tested in each tissue; see Additional file 1: Table S1) to declare transcript *i* as significantly associated with PGS_T_ or PGS_BioT_ in each sex.

### Functional and pathway enrichment analysis

We selected a list of transcripts passing a nominal cut-off of *p* < 1$\:\times\:$10^−3^ for PGS_T_ and PGS_BioT_ analyses across all the study tissues, separately for males and females (see Additional file 1: Table S3). We then used DAVID^27^ to perform gene ontology (GO), the Kyoto Encyclopedia of Genes and Genomes (KEGG)^28^, and Reactome pathway enrichment analysis. We considered the GO terms [i.e., biological processes (BP), molecular functions (MF), and cellular components (CC)] and biological pathways as significantly enriched using a false discovery rate (FDR) < 0.10.

## Results

### Independence of testosterone polygenic scores in males and females

We constructed polygenic scores for total testosterone (PGS_T_) and bioavailable testosterone (PGS_BioT_) as proxies for adult variation in these traits in the GTEx cohort. We used these PGSs to test the effect of testosterone on within-sex gene expression across tissues in the GTEx dataset. We found that the SNP weights from a specific sex have a weak predictive benefit when used in the opposite sex (e.g., the Pearson correlation of PGS_T_ in females obtained from using female versus male SNP weights was 0.17; *p* = 0.003, Fig. S1), which is consistent with previous work^18^ reporting near-zero predictive power (e.g., R² = 0.02 for females) of sex-specific PGS on testosterone levels in the opposite sex. However, the statistically significant correlation estimates in our study suggests some shared variance in testosterone genetics across sexes, despite the distinct genetic architectures – consistent with previous studies^3,6^.

Moreover, we observed a positive correlation between PGS_T_ and PGS_BioT_ in men (*r* = 0.38, 95% CI [0.31, 0.45], *p* < 0.0001) and women (*r* = 0.53, 95% CI [0.44, 0.61], *p* < 0.0001), consistent with previous work^3^, which reported higher correlation values (*r* = 0.67 in males and *r* = 0.85 in females) for raw sex hormone measures. The relatively lower correlations in our study are expected, as polygenic scores capture only a fraction of the variance in testosterone levels.

### Proportion of variance in testosterone captured by gene expression

**Fig. 1:**
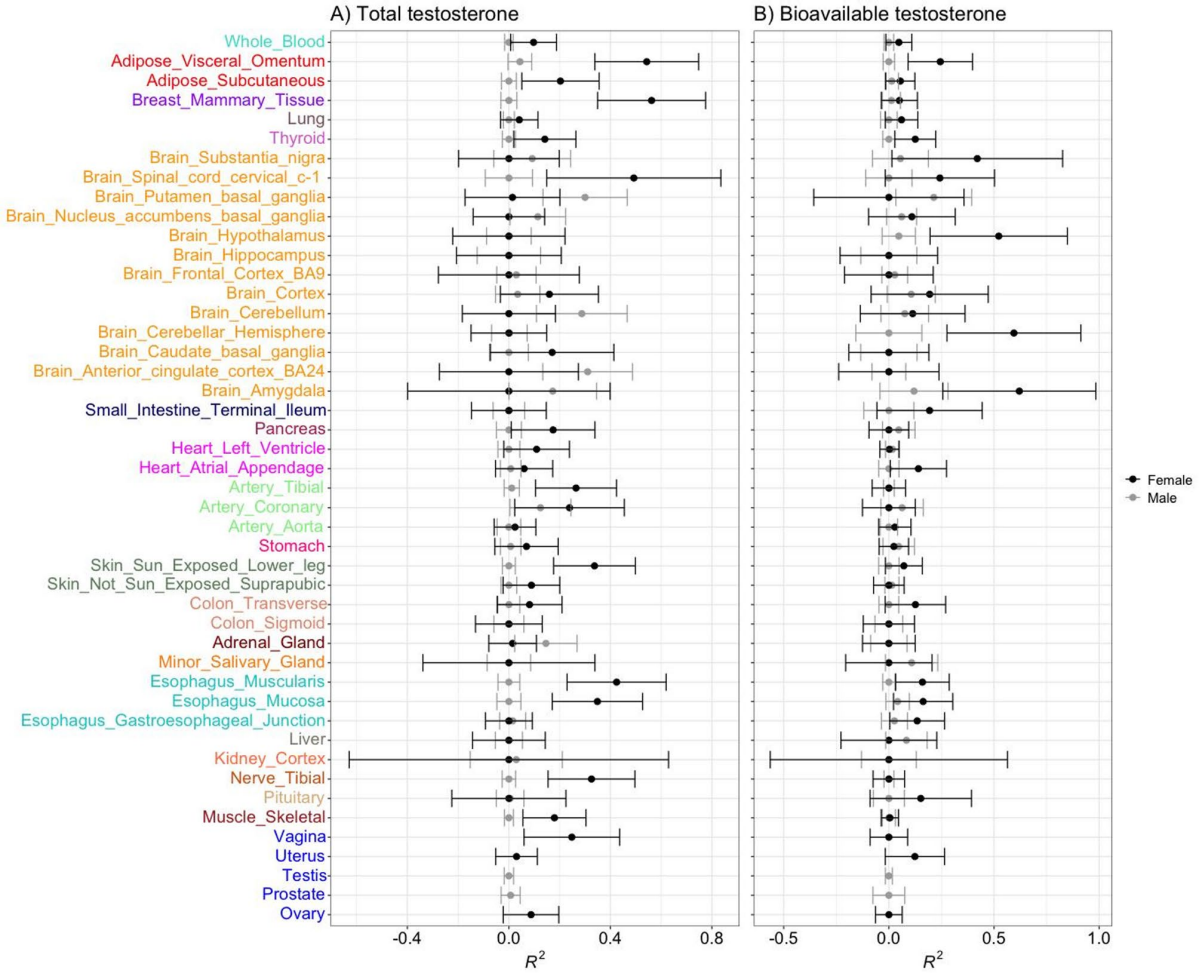
Proportion of variance ($\:{R}^{2}$) in predicted total testosterone **(B)** and bioavailable testosterone **(B)** that is associated with gene expression values in males and females across study tissues. The horizontal bars represent standard errors. Sex specific tissues are colour-coded in blue, while other colours represent related tissue groups.

To identify tissues where global gene expression is most associated with testosterone exposure in adults, we estimated the proportion of variance (denoted as R^2^) in PGS_T_ and PGS_BioT_ that can be captured when considering all gene expression transcripts in a single model (> 20,000 probes per tissue; see Additional file 1: Table S1). The R^2^ values for PGS_T_ and PGS_BioT_ differed across tissues (Fig. 1). Notably, all the variance components were estimated with large standard errors, reflecting relatively small sample sizes for gene expression data across tissues (see Additional file 1: Table S2). The estimated variance components were generally not statistically significant, except for PGS_T_ in females, which showed nominal significance (*p* < 0.05) in the adipose, breast mammary gland, esophagus (mucosa and muscularis), and skin (not-sun exposed), none of which remained significant after false discovery rate correction (FDR < 0.05) across tissues (Table S2). For PGS_T_, the R^2^ values in males ranged from 0.0 to 0.31 with a mean = 0.04, while those for females ranged from 0.0 to 0.56 with a mean = 0.12 (see Fig. 1 and Additional file 1: Table S2). In females, the gene expression in the mammary breast tissue captured the largest proportion of variance in PGS_T_, consistent with other studies showing that this tissue has relatively many sex-biased genes^29^. Other tissues with a large R^2^ value in females include the adipose visceral omentum (0.54; SE = 0.20; *p* = 0.008), brain spinal cord cervical c-1 (0.49; SE = 0.34; *p* = 0.15) and esophagus muscularies (0.42; SE = 0.19; *p* = 0.029). In contrast, and though not nominally significant, the gene expression for the brain-related tissues in males captured the largest proportion of variance in PGS_T,_ including the brain anterior cingulate cortex BA24 (0.31; SE = 0.18; *p* = 0.08), brain putamen basal ganglia (0.30; SE = 0.16; *p* = 0.071), and brain cerebellum (0.29; SE = 0.18; *p* = 0.109) (see Additional file 1: Table S2). For PGS_BioT_, the R^2^ in males ranged from 0.0 to 0.21 with a mean of 0.03, while those for females ranged from 0.0 to 0.62 with a mean = 0.10 (see Additional file 1: Table S2). Notably, none of the variance components associated with PGS_BioT_ were significant across all tissues.

### Transcriptome-wide association study (TWAS)

**Fig. 2:**
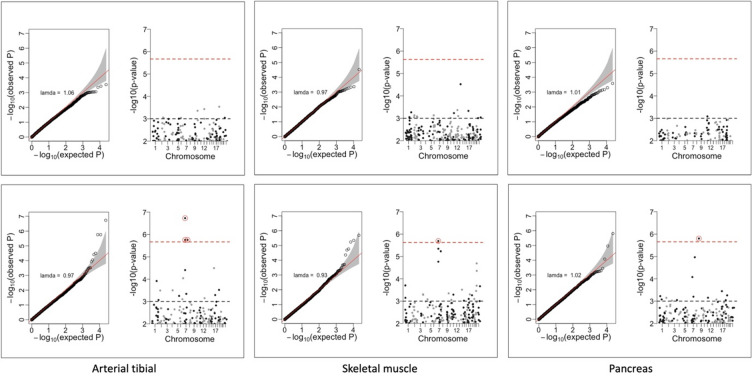
QQ and Manhattan plots of p-values for the association between polygenic scores for testosterone (PGS_T_) and gene expression in males (upper panel) and females (lower panel) across three tissues. The red dashed line indicates the Bonferroni correction threshold.

We used the mixed linear model-based omic association (MOA) approach to test the association between gene expression and predicted testosterone levels. This model has been shown to increase the power to detect associations while minimising false positives by modelling unobserved confounders^24^. Four transcripts/genes (*PSPHP1*, *NUPR1L*, *PTPRD*, and *RP11-208G20.3*) exhibited significant associations with PGS_T_ in females but not in males after a stringent Bonferroni correction for multiple testing (p < $\:2.37\times\:{10}^{-6}$) across arterial tibial, skeletal muscle, and pancreas tissue (Fig. 2; Table 1). No transcript/gene passed Bonferroni correction for PGS_BioT_ in females. In addition, no transcript/gene was significant across all male association analyses for both PGS_T_ and PGS_BioT_.

**Fig. 3:**
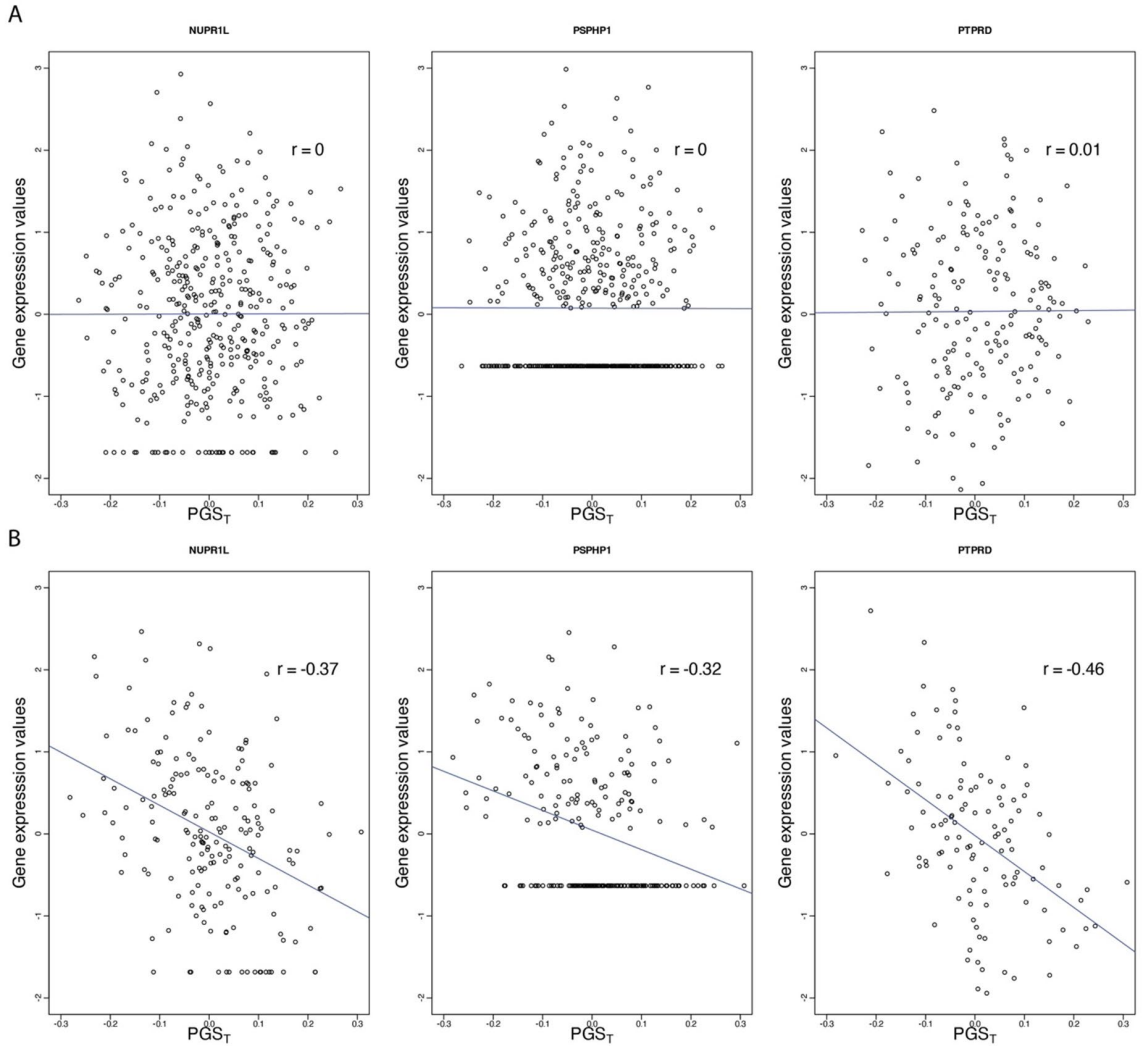
Correlation between genetically predicted testosterone (PGS_T_) and gene expression values for the *NUPR1L*, *PSPH1*, and *PTPRD* transcripts in the arterial tibial (left panel), skeletal muscle (middle panel), and pancreas (right panel) tissue in males **(A)** and females **(B)**. The identical values for *PSPH1* represent non-detected values of gene expression.

Detailed information on the most significant transcripts associated with testosterone in females is provided in Table 1. The list of all the transcripts that satisfied the nominal statistical threshold of p < $\:1.0\times\:{10}^{-3}$ for both PGS_T_ and PGS_BioT_ is given in Additional file 1: Table S1. The most significantly associated gene was the nuclear protein transcriptional regulator 1 like (*NUPR1L*) gene on chromosome 7 (p = $\:1.86\times\:{10}^{-7}$; Table 1) for the arterial tibial tissue in females for PGS_T_. This gene has a non-significant but positive estimated effect (*p* = 0.77) for PGS_T_ in the arterial tibial in males. The *NUPR1L* gene meditates many biological processes, including cell cycle, apoptosis, autophagy, and DNA repair responses. The expression of this gene in the arterial tibial is negatively correlated with testosterone in females (Pearson correlation *r* = − 0.37; Fig. 3) but not in males (*r* = 0.002).

Also, the expression of this gene was not significantly associated with PGS_BioT_ in both males and females across all the study tissues. When considering the nominal statistical threshold (p < $\:1.0\times\:{10}^{-3}$), the expression of the *NUPR1L* gene was associated with testosterone in six other tissues in females, including skin sun-exposed lower leg, esophagus muscularis, adipose subcutaneous, skeletal muscle, adipose visceral omentum, artery tibial, and mammary breast tissue (Fig. S2). In contrast, *NUPR1L* in males did not reach this statistical cut-off (p < $\:1.0\times\:{10}^{-3}$) across all the study tissues.

**Fig. 4:**
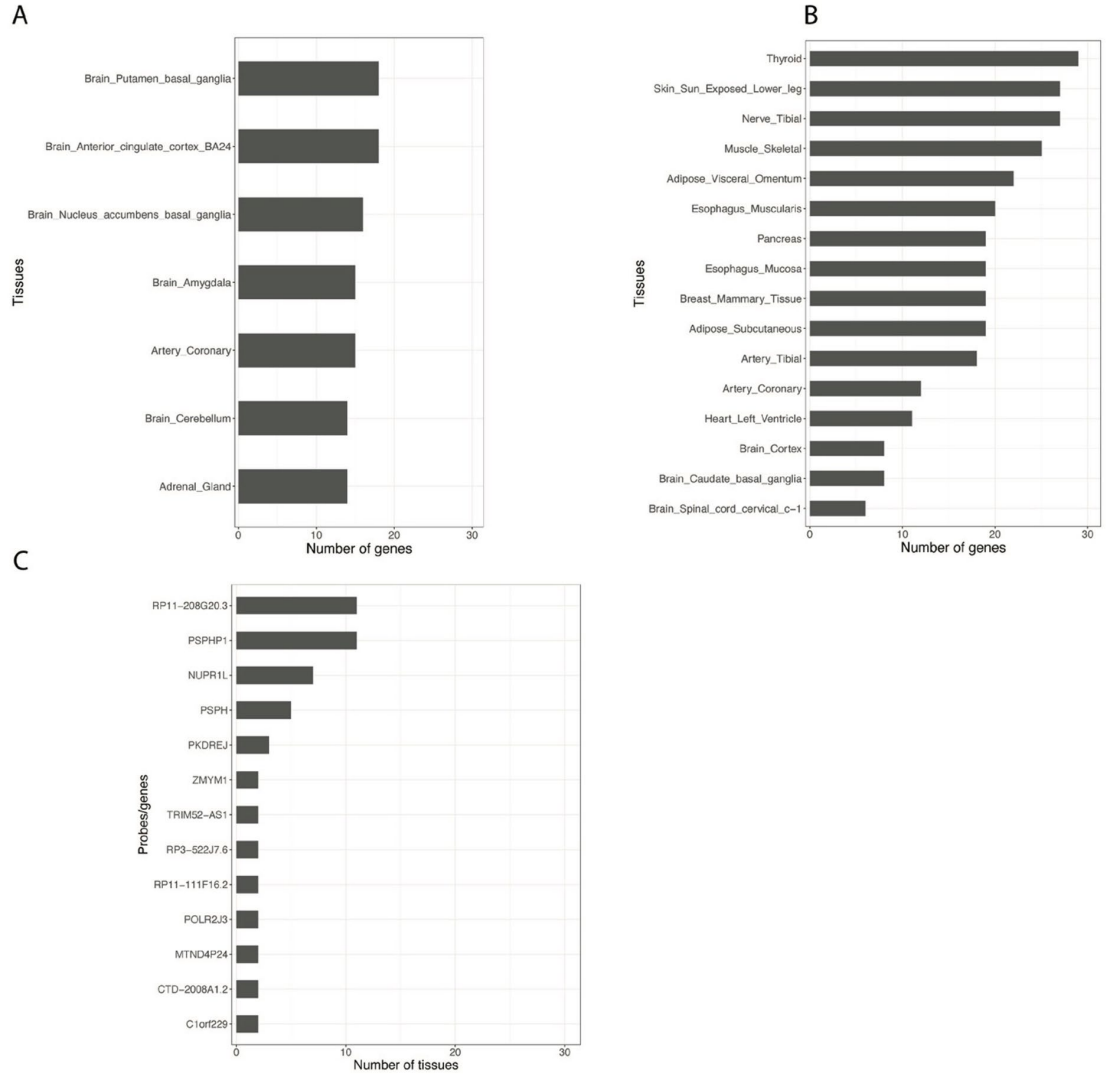
Number of transcripts/genes per tissue (A and B) which passed nominal significance (p < $\:1.0\times\:{10}^{-3})$ in males **(A)** and females **(B)**. The number of tissues in which a transcript/gene was nominally (p < $\:1.0\times\:{10}^{-3})$ significant **(C)**.

The expression of the Phosphoserine Phosphatase Pseudogene 1 (*PSPHP1*) gene (located ~ 350 kb from the *NUPR1L* gene in chromosome 7) was also significantly associated with PGS_T_ in the arterial tibial (p = $\:1.75\:\times\:\:{10}^{-6}$) and skeletal muscle (p < $\:2.03\times\:{10}^{-6}$) in females (Table S3). The Pearson correlation estimates for the PGS_T_ versus gene expression in the arterial tibial and skeletal muscle were (*r* = − 0.38) and (*r* = − 0.31), respectively. Also, around the same region of chromosome 7, the *RP11-208G20.3* transcript was significantly (p = $\:1.75\times\:{10}^{-6}$; Table S3) associated with testosterone in the arterial tibial tissue for females (Pearson correlation estimate; *r* = − 0.38). Notably, the expression values for these two transcripts in females were associated with testosterone across ten tissues at a nominal statistical threshold (p = $\:1.0\times\:{10}^{-3}$; see Additional file 1: Table S3 and Fig. 4). In males, these transcripts were not nominally significant across all study tissues.

The expression of the Protein Tyrosine Phosphatase Receptor Type D (*PTPRD*; chromosome 9 ~ 83 Mb) gene in the pancreas tissue was significantly negatively correlated with PGS_T_ only in females (p = $\:1.57\times\:\:{10}^{-6}$). Pearson correlation estimate (*r* = − 0.46; Fig. 3). This gene encodes a protein belonging to the protein tyrosine phosphatase (PTP) family, which mediates biological processes, including cell growth, differentiation, mitotic cycle, and oncogenic transformation.

**Table 1:** Top significant transcripts from the mixed linear model-based omic association (MOA) analyses in females.

Chr	Transcript	Position	Gene	*P*-value	Tissue	Trait
7	ENSG00000226278*	55,832,490	*PSPHP1*	1.75 $\:\times\:{10}^{-6}$	Artery tibial	PGS_T_
7	ENSG00000226278*	55,832,490	*PSPHP1*	2.03 $\:\times\:{10}^{-6}$	Muscle skeletal	PGS_T_
7	ENSG00000226278	55,832,490	*PSPHP1*	4.34 $\:\times\:{10}^{-6}$	Adipose visceral omentum	PGS_T_
7	ENSG00000146733	56,078,744	*PSPH*	1.72 $\:\times\:{10}^{-5}$	Muscle skeletal	PGS_T_
7	ENSG00000185290*	56,182,374	*NUPR1L*	1.86 $\:\times\:{10}^{-7}$	Artery tibial	PGS_T_
7	ENSG00000185290	56,182,374	*NUPR1L*	4.51 $\:\times\:{10}^{-6}$	Muscle skeletal	PGS_T_
7	ENSG00000270405*	152,063,848	*RP11-208G20.3*	1.75 $\:\times\:{10}^{-6}$	Artery tibial	PGS_T_
7	ENSG00000270405	152,063,848	*RP11-208G20.3*	5.86 $\:\times\:{10}^{-6}$	Muscle skeletal	PGS_T_
7	ENSG00000270405	152,063,848	*RP11-208G20.3*	1.09 $\:\times\:{10}^{-5}$	Pancreas	PGS_T_
7	ENSG00000270405	152,063,848	*RP11-208G20.3*	2.43 $\:\times\:{10}^{-5}$	Esophagus mucosa	PGS_T_
8	ENSG00000184428	144,386,554	*TOP1MT*	1.20 $\:\times\:{10}^{-5}$	Whole blood	PGS_T_
8	ENSG00000254338	144,499,849	*RP11-909N17.3*	1.13 $\:\times\:{10}^{-5}$	Nerve tibial	PGS_T_
9	ENSG00000228322	4,299,390	*GLIS3-AS2*	2.66 $\:\times\:{10}^{-5}$	Thyroid	PGS_T_
9	ENSG00000153707*	8,314,246	*PTPRD*	1.57 $\:\times\:{10}^{-6}$	Pancreas	PGS_T_
10	ENSG00000166917	133,246,478	*MIR202HG*	2.19 $\:\times\:{10}^{-5}$	Breast mammary tissue	PGS_T_
16	ENSG00000250251	15,219,099	*PKD1P6*	2.34 $\:\times\:{10}^{-5}$	Adipose visceral omentum	PGS_BioT_
20	ENSG00000132801	44,486,256	*ZSWIM3*	1.39 $\:\times\:{10}^{-5}$	Thyroid	PGS_T_
22	ENSG00000130943	46,651,560	*PKDREJ*	6.93 $\:\times\:{10}^{-6}$	Nerve tibial	PGS_T_
22	ENSG00000130943	46,651,560	*PKDREJ*	2.05 $\:\times\:{10}^{-5}$	Muscle skeletal	PGS_T_
X	ENSG00000275070	129,796,491	*Metazoa_SRP*	8.98 $\:\times\:{10}^{-6}$	Nerve tibial	PGS_T_

*Genes/transcripts that survived stringent Bonferroni correction.

### Gene enrichment analysis

We used the DAVID functional annotation tool to explore enriched biological features for the set of transcripts that passed nominal significance (p < $\:1.0\times\:{10}^{-3}$; see Additional file 1: Table S3) in our association analyses. Notably, most of the nominally significant genes in males (Fig. 4A) were found in the brain-related tissues putamen basal ganglia and anterior cingulate cortex BA24 (*N* = 18). In contrast, the largest number of nominally significant genes in females were found in the thyroid tissue (*N* = 29), followed by skin (sun-exposed lower leg; *N* = 27) and nerve tibial (*N* = 27) (Fig. 4B).

The gene ontology terms and biological pathways for gene transcripts with p-values < 0.05 in males and females for PGS_T_ and PGS_BioT_ are provided in Additional file 1: Table S4. The *regulation of T cell proliferation* (GO:0042129; *p* = 0.005) and *cytokine-mediated signaling pathway* (GO:0019221; *p* = 0.02) (see Additional file 1: Table S4) are the gene ontology terms with the smallest p-values for PGS_T_ in females. Similarly, the most enriched pathways in females associated with PGS_T_ included *Ras activation upon Ca2 + influx through NMDA receptor* (*p* = 0.009) and *Negative regulation of NMDA receptor-mediated neuronal transmission* (*p* = 0.01; see Additional file 1: Table S4*)*. On the other hand, the most enriched GO terms in males for PGS_T_ are related to the *ATPase activity* (GO:0016887; *p* = 0.006) and the *regulation of transcription from RNA polymerase II promoter in response to stress* (GO:0043618; *p* = 0.009) (see Additional file 1: Table S4). In addition, the *Interleukin-4 and Interleukin-13 signaling* was the top enriched pathway in males for genes associated with PGS_T_ versus gene expression across all the study tissues (see Additional file 1: Table S4). As for PGS_T_, we also found that genes associated with PGS_BioT_ versus gene expression were weakly enriched for various biological features (see Additional file 1: Table S4).

## Discussion

In this study, we used publicly available gene expression data from the GTEx consortium^30^ and large published sex-specific GWAS summary statistics^3,6^ to (1) quantify the strength of association between testosterone and gene expression measures within each sex across human tissues and (2) identify sex-specific testosterone-responsive genes/transcripts in different tissues. Although we could not observe statistically significant associations across most tissues, we did find suggestive associations (*p* < 0.05) between testosterone and gene expression in female in some tissues including, mammary breast and adipose (visceral omentum) and esophagus (mascularies). However, no statistically significant associations were observed in males across any tissues for both testosterone and bioavailable testosterone. In addition, we identified four testosterone-responsive transcripts/genes (*NUPR1L*, *PTPRD*, *PSPHP1*, and *RP11-208G20.3*) in females but not males across the skeletal muscle, tibial artery, and pancreas tissue. These findings highlight molecular features potentially contributing to sex-specific patterns in human traits and diseases.

The SNP heritability estimate for total testosterone used in our study is higher in men (0.17) than in women (0.13)^3^. We found, in general, higher captured variance of testosterone by gene expressions in women than in men (though not mostly significant within each sex). One possible reason could be attributed to the statistical scaling effects since we used genomically predicted testosterone (PGSs) (instead of direct measures of testosterone) in our correlation analyses, which are associated with residual errors. The proportion of variance for PGS_T_ and PGS_BioT_ explained by gene expression differed considerably across tissues (Fig. 1). Although the reason for this difference is unclear, it appears that PGS_T_ is associated with gene expression variance in tissues such as the breast mammary gland, adipose, esophagus, and skin, particularly in females, whereas PGS_BioT_ does not show similar associations. Notably, while bioavailable testosterone is often regarded as a more biologically active form of testosterone^9,11^, our results did not show a stronger association between PGS_BioT_ and gene expression compared to PGS_T_. The higher proportion of testosterone variance captured by expression values for some tissues in females than in males agrees with others e.g.,^31^, which found consistently higher heritability estimates in females than males for traits exhibiting sex differences. It is not surprising that the gene expression values in the mammary breast tissue captured the largest proportion of testosterone variance in females (0.56 ± 0.21; *p* = 0.008) than males (0.0 ± 0.01; *p* = 1.0) since this tissue exhibit sex-biased expression pattern^32^ with many differentially regulated genes^29^. Also, the adipose (visceral omentum) tissue captured a large proportion of testosterone variance in females (*p* = 0.008) but not males (*p* = 0.36), consistent with a strong sex-biased expression pattern associated with adipose tissue in previous reports^29,32^.

Some genes/transcripts associated with gene expression in our study have been linked with specific human traits and disease endpoints. For example, the variants in/near the *PTPRD* locus are associated with puberty timing (age at menarche) in the European^33^ and Japanese^34^ female populations. In addition, variants in the *PTPRD* are associated with neurological pathologies for Alzheimer’s disease (AD)^35^– a disease that is more prevalent in women than men^36^. The expression of *NUPR1L* (also called *NUPR2*) has not been reported to function in a sex-biased manner. However, evidence suggests that the *NUPR1L* down-regulates its paralog *NUPR1* – a gene that is upregulated during an acute phase of pancreatitis^37,38^. Research shows that the PSPH family genes involved in L-serine biosynthesis are associated with neurodegenerative disorders and function in a sex-specific manner in brain-related tissues^39^. In this study, we did not detect significant associations in the brain tissues in both men and women.

Our study suffers from several limitations. First, the small sample sizes for the gene expression measures across study tissues limit the statistical power to detect associations in this study. The largest gene expression measures in this study were from the skeletal muscle in males (*N* = 469) and females (*N* = 237). Much larger sample sizes will be required to unravel within-sex genetic differences^40^. Furthermore, we did not have access to an independent sample to evaluate the predictive power of our polygenic scores. However^8^, demonstrated that PGS for total testosterone in the UK Biobank data explained 4.3% (males) and 1.9% (females) of the variance in testosterone levels in an independent cohort of Young Finns, while PGS for free testosterone explained 1.1% (males) and 1.8% (females) of the variance. Second, our study samples (GTEx participants) were mostly older individuals of European ancestry; therefore, the results are not generalizable to other populations. Our findings in females reflect post-menopausal level of circulating testosterone, given the age of the GTEx cohort (mostly > 50 years) and the UK Biobank participants (median age ~ 56 years) used for predicting polygenic scores for testosterone in the GTEx dataset. Moreover, testosterone declines with aging in both males and females^41^, meaning our results do not represent testosterone levels in younger populations since the samples were mainly from the elderly population (> 50 years). Third, testosterone regulates gene expression through androgen receptors, but its levels are also influenced by endocrine signaling pathways^6,42^. As such, our models examined associations between gene expression profiles and genetically predicted testosterone levels, but did not test the reverse—that is, the causal effect of testosterone on gene expression. Future work could explore causal relationships between these traits using more appropriate tools, such as Mendelian randomization. Fourth, it is possible that sex-biased genes manifest at the single-cell level, and we may have failed to observe sex-specific associations because we used bulk gene expression data. As such, the heterogeneity of cell types in the bulk expression data and other environmental factors may have confounded our discoveries. Since sex hormones play a role in epigenetic modifications^29,43^, it would be worth extending our results to other omic datasets, such as the DNA methylation and proteomic measurements, to gain further insights into the molecular basis underlying sex-specific differences in human traits. Finally, our findings should be interpreted with caution, until replication of suggestive hits is performed in an independent cohort. Currently, there is a lack of relevant large cohorts with expression data available for replication and follow-up confirmation.

## Supplementary Information

Below is the link to the electronic supplementary material.

Supplementary Material 1

Supplementary Material 2

## Data Availability

We used publicly available gene expression data from GTEx project ([https://www.gtexportal.org/home/]) and the UK Biobank summary statistics from two published studies (DOI: (https:/doi.org/10.7554/elife.58615) and (https:/doi.org/10.1038/s41591-020-0751-5)). All the data supporting our conclusions are included in the article and supplementary files. The scripts for running analysis and generating visualizations are available at: [https://github.com/evanskip1-88/gene_expression_vs_testosterone]. GENECODE: https://www.gencodegenes.org/human/release_26.html.

## Abbreviations

AD: Alzheimer’s disease
BioT: Bioavailable testosterone
BP: Biological processes
GWAS: Genome wide associations study
GTEx: Genotype–Tissue Expression project
GO: Gene ontology
LD: Linkage disequilibrium
MF: Molecular functions
MOA: Mixed–linear–model omic association
OREML: Omics restricted maximum likelihood analyses
OSCA: OmicS–data–based Complex trait Analysis
PEER: Probabilistic estimation of expression residuals
PGS: Polygenic scores
PGS_BioT_: Polygenic scores for predicted bioavailable testosterone
PGS_T_: Polygenic scores for predicted total testosterone
SHBG: Sex hormone–binding globulin
SNP: Single nucleotide polymorphism
TMM: Trimmed Mean of M–values
TPM: Transcript per million
TWAS: Transcriptome–wide association study
