# Supplementary material for: Effects of testosterone on gene expression in males and females across 40 human tissues
Source: Sci Rep. 2026 Feb 23;16:10223. doi: 10.1038/s41598-026-40863-2 (PMC13031714; doi:10.1038/s41598-026-40863-2)
Supplement: Supplementary file 1 — Supplementary Material 1 [file 41598_2026_40863_MOESM1_ESM.docx]

# Effects of testosterone on gene expression in males and females across 40 human tissues

**Supplementary figures**


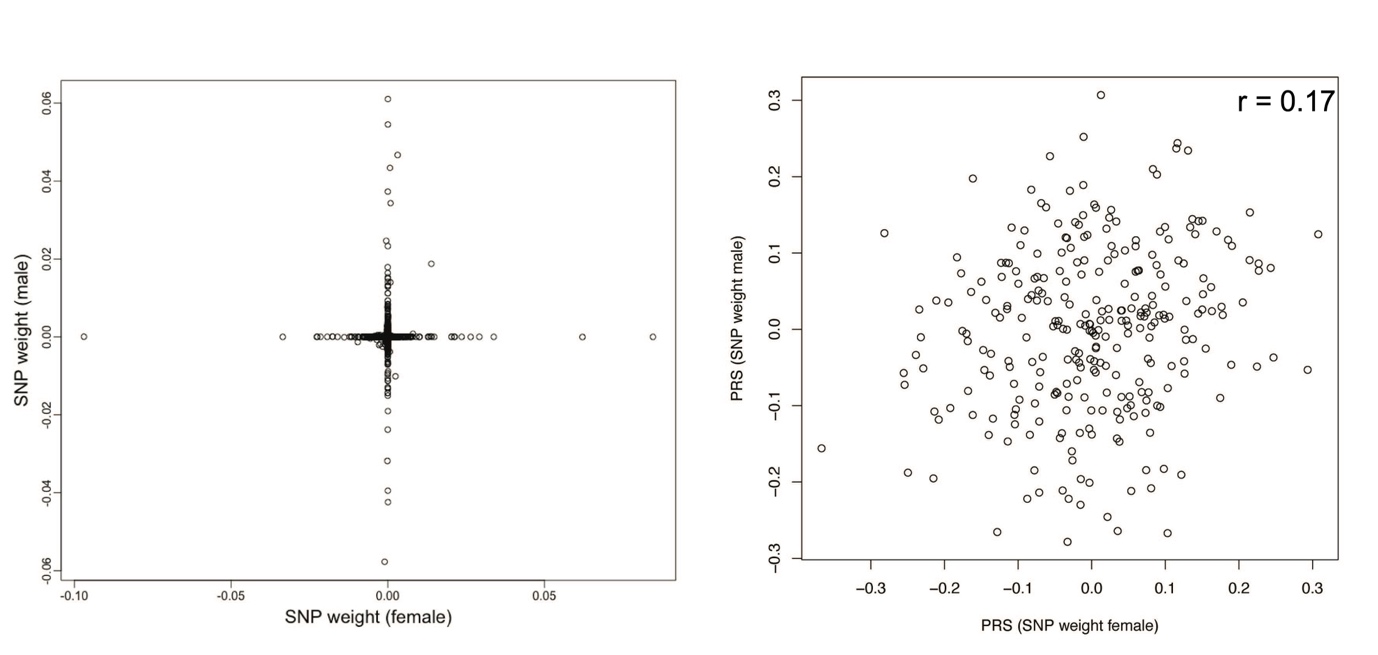


**Figure S1:** SNP weights for the effect alleles estimated from the summary statistics for testosterone (left plot). Correlation between polygenic scores for testosterone in females constructed based on the same versus opposite sex SNP weights (right plot).


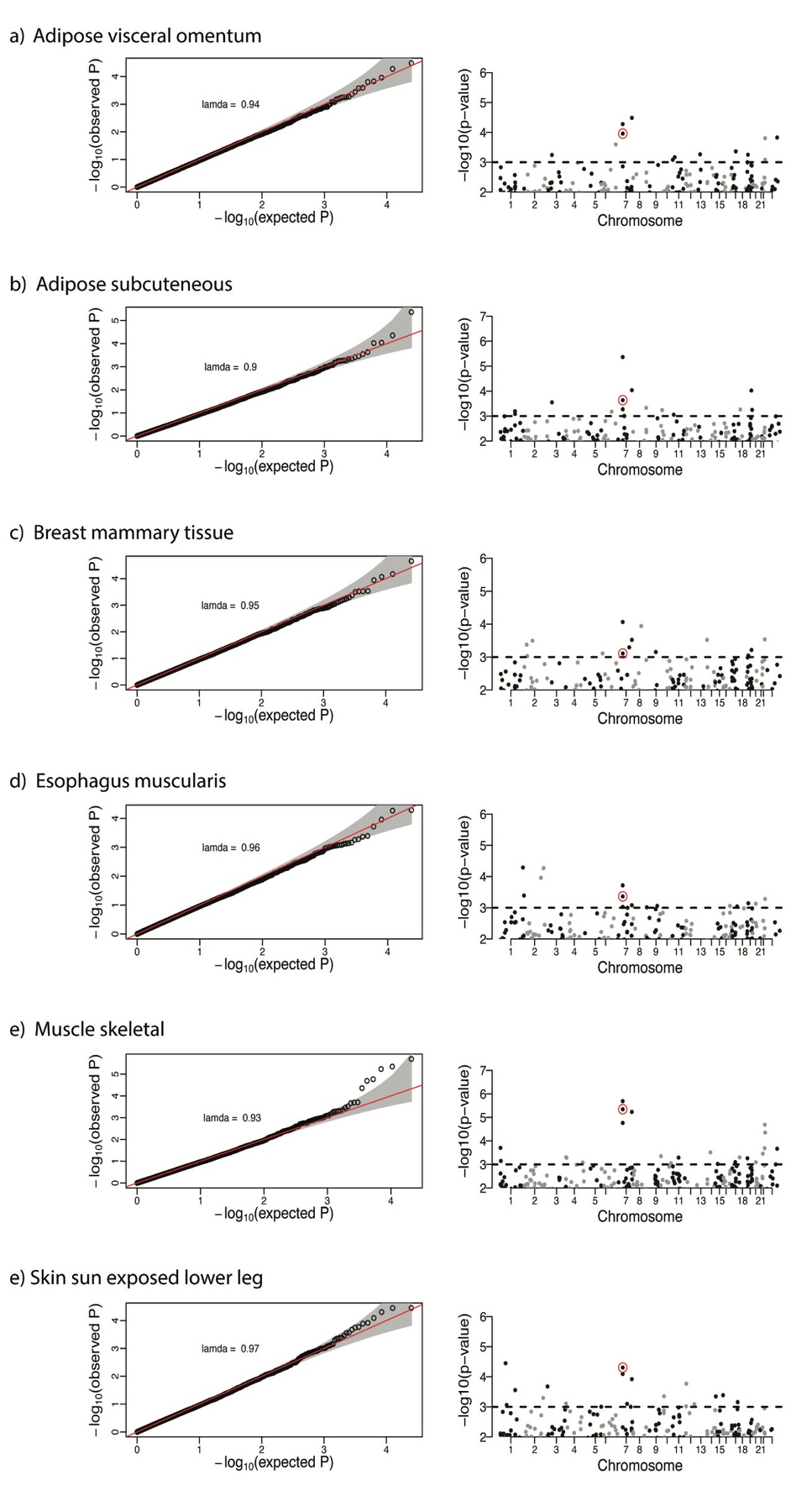


**Figure S2:** QQ and Manhattan plots of p-values for the association between testosterone and gene expression in females across six tissues. The highlighted red point on the Manhattan plot is the *NUPR1L* gene.
